# Acoustic Presence of Cetaceans in the Miaodao Archipelago, China

**DOI:** 10.3390/ani13081306

**Published:** 2023-04-11

**Authors:** Zhaolong Cheng, Yongtao Li, Matthew Keith Pine, Xiaoling Wan, Tao Zuo, Mingxiang Niu, Jun Wang

**Affiliations:** 1 Yellow Sea Fisheries Research Institute, Chinese Academy of Fishery Sciences, Qingdao 266071, China; 2 Laboratory for Marine Ecology and Environmental Science, Pilot National Laboratory for Marine Science and Technology (Qingdao), Qingdao 266237, China; 3 Department of Biology, University of Victoria, Victoria, BC V8P 5C2, Canada; 4 School of Animal Science and Nutritional Engineering, Wuhan Polytechnic University, Wuhan 430023, China

**Keywords:** passive acoustic monitoring, species diversity, distribution, conservation

## Abstract

**Simple Summary:**

Little effort has been made to conserve cetaceans in the Miaodao Archipelago, which is hindered by a lack of baseline data on their species and distribution patterns. Using a passive acoustic monitoring technique, we found a decrease in cetacean diversity; the East Asian finless porpoise is the sole cetacean species that can be reliability detected in this area, and their distribution exhibits seasonally patterns. Further research and conservation measures are needed to protect cetaceans in this area.

**Abstract:**

Once an important cetacean habitat, the Miaodao Archipelago has been altered by human-induced disturbances over several decades. While cetacean diversity is known to have decreased, no recent data on species diversity around Miaodao are known to exist. Capitalizing on the high vocal activity of cetaceans, three passive acoustic surveys, including towed and stationary types, were undertaken to detect the presence of species-specific vocalizations in May 2021, October 2021, and July 2022, as most cetacean sightings occurred during May and August in recent years. The results revealed that the East Asian finless porpoise is the sole cetacean species that can be reliably observed around the archipelago, as no other species were detected. The acoustic data also revealed potentially clumped distributions of finless porpoises with some seasonal variation. While not acoustically detected during any of the surveys, humpback whales, minke whales, and killer whales have been visually sighted in the region. The lack of acoustic detection of these species suggests that they are likely to be temporary visitors to the region, or at least exhibit strong seasonality in their presence within the region. These new data provide the latest snapshot of cetacean presence around the Miaodao Archipelago that can help inform future research and conservation.

## 1. Introduction

The Miaodao Archipelago is made up of 32 small islands and is located at the intersection of the Yellow Sea and the Bohai Sea, between the Shandong and Liaodong Peninsulas. This region is characterized by a temperate, semi-humid continental and ocean climate, and supports economically important commercial fisheries and agriculture [1]. Historical whaling and stranding records show that the Miaodao Archipelago is utilized by both mysticetes and odontocetes, including four baleen species (humpback whale (*Megaptera novaeangliae*), minke whale (*Balaenoptera acutorostrata*), fin whale (*Balaenoptera physalus*), and gray whale (*Eschrichtius robustus*)) and five odontocete species (killer whale (*Orcinus orca*), false killer whale (*Pseudorca crassidens*), short-beaked common dolphin (*Delphinus delphis*), common bottlenose dolphin (*Tursiops truncatus*), and the marine subspecies of narrow-ridged finless porpoise (*Neophocaena asiaeorientalis*)-the East Asian finless porpoise (*N. a. sunameri*)) [2]. However, cetacean species in this region have been suffering sharp declines in population size due to detrimental influences from human activities, such as commercial whaling, fishery bycatch, chemical and noise pollution, and overfishing [3]. Based on the recent sighting records, only two baleen and two odontocete species have been found in these waters (such as humpback whale, http://www.jiaodong.net/travel/system/2018/05/07/013670986.shtml (accessed on 9 April 2023); minke whale, https://ishare.ifeng.com/c/s/7nzD8FqWGIS (accessed on 9 April 2023); killer whale, https://www.sohu.com/a/329178594_120044938 (accessed on 9 April 2023), and https://v.youku.com/v_show/id_XMzk0MTk4NjUwNA==.html (accessed on 9 April 2023); East Asian finless porpoise, https://www.toutiao.com/article/6663770826057187844/?wid=1660867925355 (accessed on 9 April 2023), https://sjb.qlwb.com.cn/qlwb/content/20220526/ArticelA01003FM.htm (accessed on 9 April 2023), and http://w.dzwww.com/p/5172554.html (accessed on 9 April 2023)). All of the four species are listed in the National Key Protected Animals in the newly modified list of key protected wild animals in China in 2021, of which humpback whale, minke whale, and killer whale are Grade 1 protected animals and the East Asian finless porpoise is a Grade 2 protected animal, meaning it is critically necessary for them to be protected. There is thus a need to further understand basic information on the population of these species within and around the Miaodao Archipelago, such as their distribution patterns and dynamics, and behaviors, so as to push for appropriate conservation practices.

Despite this, little scientific attention has been given to study the cetacean presence within the Miaodao Archipelago. A key reason may be that effective monitoring of these species is challenging. The classical approach to monitoring cetaceans, being visual-based methodologies, continues to provide valuable information on cetaceans, including their abundance and distribution patterns. However, despite their benefits, visual surveys are expensive (particularly vessel-based surveys), time consuming, labor intensive, and limited to good weather and visibility conditions [4]. In addition, some species, in particular the finless porpoise, can sometimes be missed by observers due to their fast swimming and lack of dorsal fin [5]. The passive acoustic method counters these limitations and has thus become a fast-evolving tool in marine mammal monitoring. Passive acoustic monitoring (PAM) is cost-effective and has been widely used in cetacean studies [5,6,7]. Cetaceans rely heavily on their vocalizations in order to adapt to their underwater environment, making them particularly suitable for PAM approaches [6].

Humpback whales are commonly known for their singing, made up of repeated series of complex vocalizations [8]. They also produce vast repertoires of non-song social calls. Males sing during the breeding season, whereas both males and females produce social calls when in groups of three or more adults [9]. The fundamental frequency of sounds produced by humpback whales ranges between tens of Hz and 4 kHz, with higher harmonics extending beyond 24 kHz [10]. Minke whales in the North Pacific produce unique vocalizations (commonly referred to as boings) during their breeding season from fall to spring [11]. The fundamental frequencies of boings ranged between 1 and 1.8 kHz, with harmonics extending to approximately 9 kHz [12]. Killer whales produce broad-band echolocation clicks up to 85 kHz, whistles between 1.5 kHz and 18 kHz, and pulse calls that appear tonal but are a rapid series of pulses with peak energies between 1 and 6 kHz [13,14,15]. East Asian finless porpoises produce narrowband high-frequency echolocation clicks well over 100 kHz that can be easily identified from other bio- and abiotic sounds [16].

Considering that cetacean sightings occur mostly during May and August, we investigated the acoustic presence of the species and distribution patterns of cetaceans in the Miaodao Archipelago using a PAM system in May 2021, October 2021, and July 2022 in this study. As all of the potentially existing species in this region are considered endangered and data deficient, the information provided here could be helpful for future conservation efforts.

## 2. Materials and Methods

### 2.1. Data Collection

**Figure 1:**
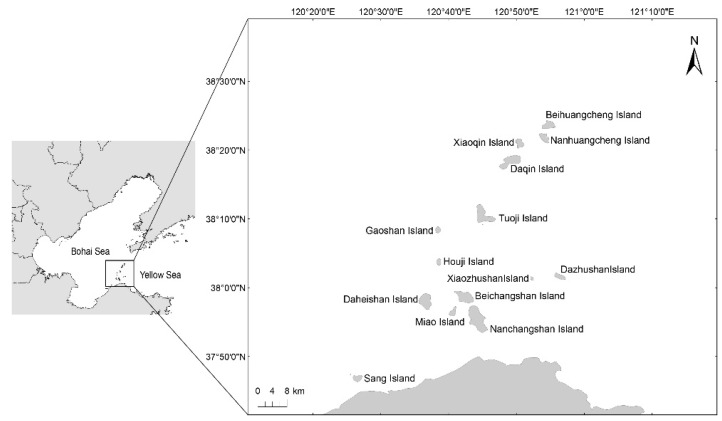
Map of the passive acoustic monitoring area where the humpback whale, minke whale, killer whale, and East Asian finless porpoise may be encountered.

Data were collected using a SoundTrap 300HF (Ocean Instruments, Auckland, New Zealand) recorder that was set to record continuously at a 576 kHz sampling rate in the Miaodao Archipelago (Figure 1).

To cover the study area evenly, towed PAM survey transects were spaced at 10 km. A 26 m long fishing vessel (at approximately 12 km/h) was used to tow the recorder 100 m behind the boat using a nylon rope. An iron bar (approximately 300 g) was affixed to the front of the recorder, and five floats were also attached to the nylon rope to suspend the recorder in the seawater without touching the sea bed. A time-stamped GPS receiver (Garmin GPSMAP 639sc; Garmin International Ltd., Olathe, KS, USA) was used to track the survey routes.

Stationary PAM surveys were conducted during the nighttime of the towed PAM surveys when the fishing vessel was anchored and the engine was turned off. The recorder was attached with plastic bands to a rope, and was deployed vertically into the water at 2.5 m depth using a 7 kg weight.

### 2.2. Data Analysis

Acoustic recordings were downloaded and converted to WAV files using SoundTrap host software (Ocean Instruments, Auckland, New Zealand). The WAV files were then uploaded to Raven Pro 1.6 software (the Cornell Lab of Ornithology, Ithaca, NY, USA) to manually identify potential cetacean vocalizations.

To pick out the sound of humpback whales and minke whales, acoustic recordings were inspected in the range of 0–5 kHz with the following parameters: window type: Hanning; window size: 10,000; 3 dB filter bandwidth: 82.8 Hz; time grid overlap: 50%; hop size: 8.68 ms; frequency grid DFT size: 16,384; grid spacing: 35.2 Hz. To locate the sounds from the killer whale and East Asian finless porpoises, acoustic recordings were inspected in full bandwidth with the following parameters: window type: Hanning; window size: 2048; 3 dB filter bandwidth: 405 Hz; time grid overlap: 50%; hop size: 1.78 ms; frequency grid DFT size: 2048; grid spacing: 281 Hz.

A detection was defined as a series of vocalizations within 5 min following the last vocalization. In towed PAM, the acoustic detection density was calculated by dividing the survey distance into the numbers of detection.

## 3. Results

**Figure 2:**
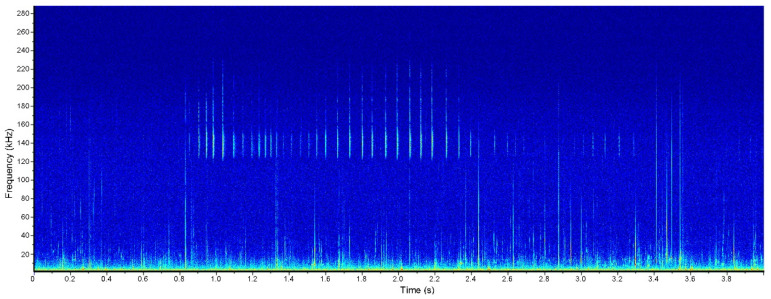
Spectrogram of the narrow band high frequency clicks recorded from the East Asian finless porpoises in Miaodao Archipelago waters (hanning; window size: 2048; 3 dB filter bandwidth: 405 Hz; time grid overlap: 50%; hop size: 1.78 ms; frequency grid DFT size: 2048; grid spacing: 281 Hz).

Surveys were conducted in May 2021, October 2021, and July 2022. In towed PAM, the recorder was towed 356.49 km in 30.08 h, 423.52 km in 32.58 h, and 504.76 km in 44.75 h, respectively. In stationary PAM, the recorder was deployed for 124.62 h, 87.85 h, and 103.28 h, respectively. No humpback whale, minke whale, or killer whale vocalizations were detected. Only narrow band high frequency clicks from the East Asian finless porpoises were detected (Figure 2).

**Figure 3:**
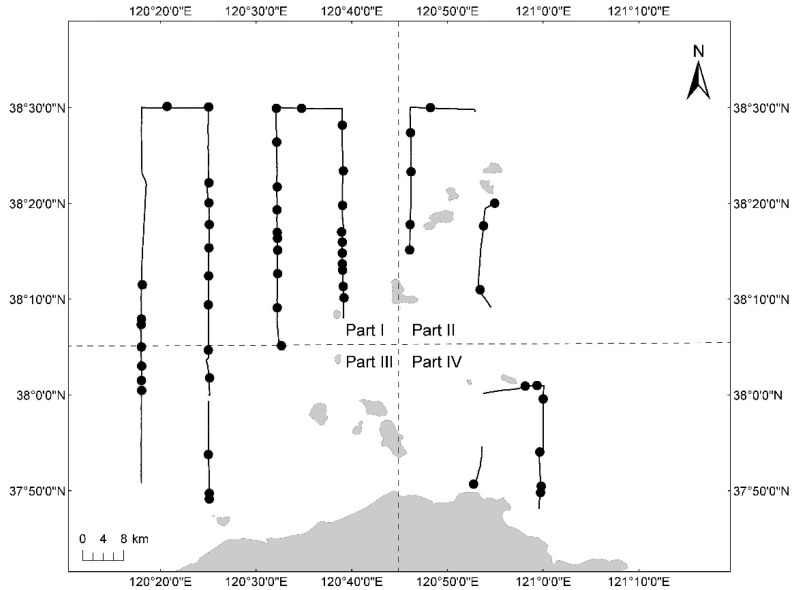
Survey routes (black line) and acoustic detection location (black dot) for the East Asian finless porpoises in Miaodao Archipelago waters in May 2021.

**Figure 4:**
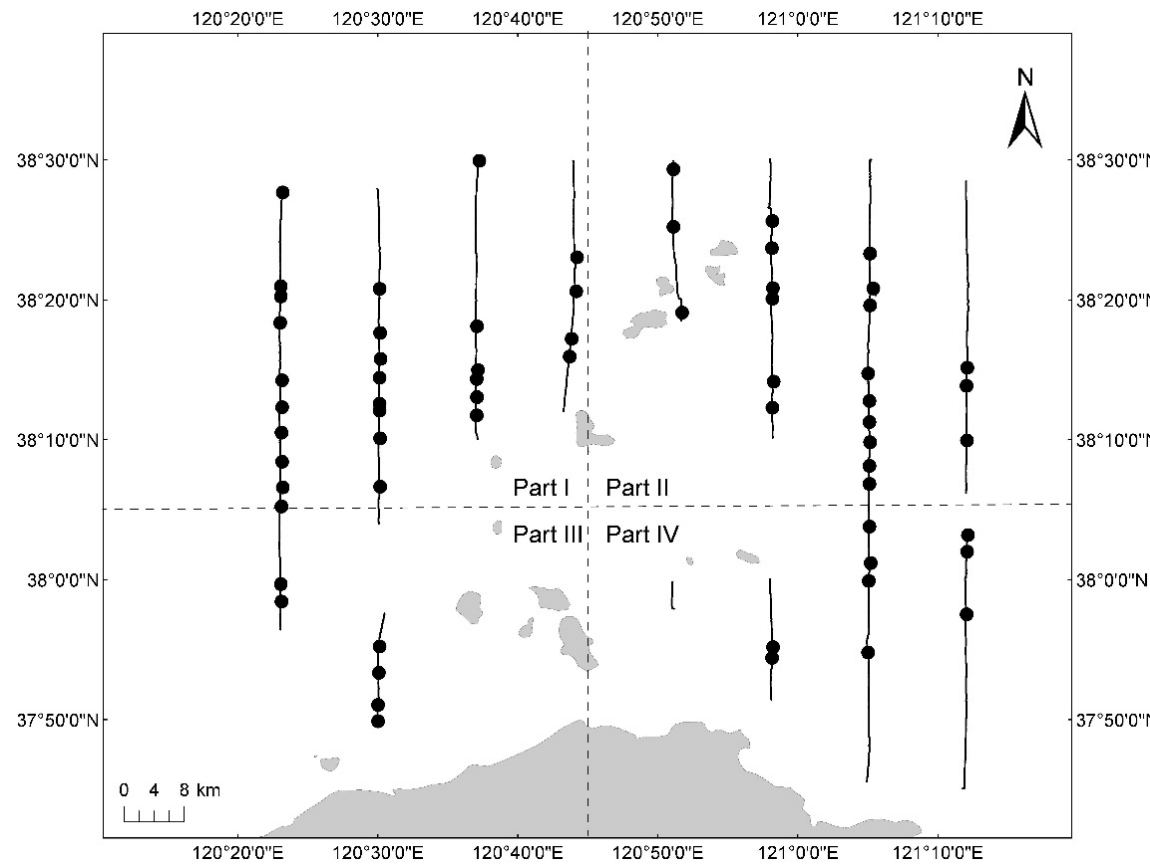
Survey routes (black line) and acoustic detection location (black dot) for the East Asian finless porpoises in Miaodao Archipelago waters in October 2021.

In towed PAM, 169 East Asian finless porpoise detections (56 in May 2021, 64 in October 2021, and 49 in July 2022) were found, which were throughout the Miaodao Archipelago, but displayed a seasonal distribution pattern. Dividing the survey area into four parts by N38°05′ and E120°45′, the details of the porpoise detection, survey distance, and porpoise detection density in each part are listed in Table 1. Survey routes and detection locations are shown in Figure 3, Figure 4andFigure 5.

**Figure 6:**
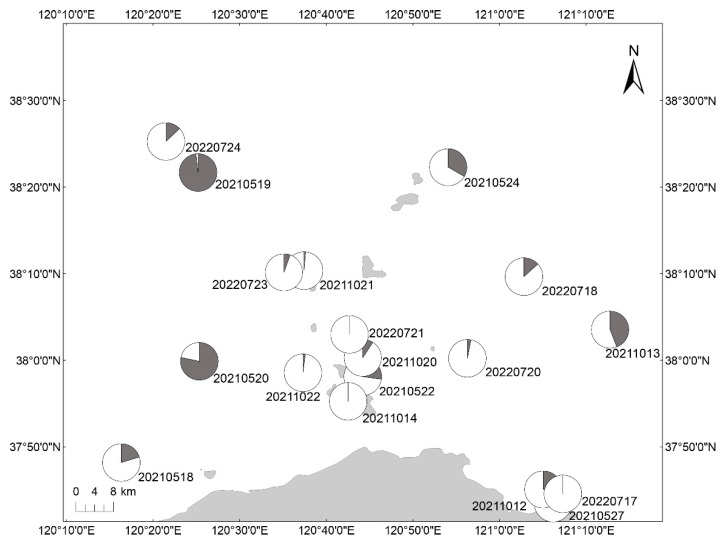
Map showing the proportions of the duration of porpoise acoustic detection in each site (dark in pie charts) in Miaodao Archipelago waters in May 2021, October 2021, and July 2022.

In stationary PAM, 99 East Asian finless porpoise detections (54 in May 2021, 24 in October 2021, and 21 in July 2022) were found. The duration of the porpoise acoustic detection recorded from each site is showed in Table 2 and Figure 6. In summary, the proportions of times in which porpoises were detected were 32.14% in May 2021, 11.64% in October 2021, and 5.02% in July 2022.

## 4. Discussion

Based on historical records and recent citizen information, it was expected that at least vocalizations from humpback whale, minke whale, killer whale, and East Asian finless porpoise would be found in our recordings. However, in the present study, only narrow band high frequency clicks, i.e., sounds produced by East Asian finless porpoise, were detected.

Our results suggest that the diversity of cetaceans around the Miaodao Archipelago waters have declined: only four species were sighted in recent years, and only East Asian finless porpoises were detected in our recordings. Cheng et al. [3] suggest that whaling (stopped since 1980 when China joined the International Whaling Commission), food deficiency caused by overfishing, bycatch, and environmental pollution were their main threats. The minke whales in Miaodao Archipelago were previously believed to originate from Japan, while the humpback whales were thought to come from Sea of Okhotsk, and killer whales were believed to be from the Chukchi Peninsula [17]. Their sightings in Miaodao Archipelago mostly occurred between May and August, when several fish species migrate through this area [18]. Notwithstanding, the precise origins and the motivations behind some patterns still require further study.

The present study further suggests that the Miaodao Archipelago is a vital habitat for the East Asian finless porpoise, concurring with previous data from Cheng et al. [3] who obtained baseline information of the East Asian finless porpoises in south Bohai Sea using local ecological knowledge. In addition, the present study updates the baseline distribution information for the porpoise in Miaodao Archipelago: in May and October, their distribution was relative even, as the detection rates in all four parts were above or equal 0.10 per km; however, in July, a heterogeneous distribution pattern was found, and their detections were concentrated in west part of the Miaodao Archipelago, i.e., part I and part III in the present study. Human disturbance and prey spatio-temporal distribution are the key factors influencing the fine-scale habitat use in cetacean [19,20]. In this study, the porpoises spent more time in areas distant from islands and lands. It is possible that porpoises preferred these habitats owing to the low level of disturbance from human activities. The distribution and movement patterns of East Asian finless porpoises in the adjacent waters were found to be closely correlated with prey [5,21]. Seasonal porpoise distribution variation in the present study may indicate the area changes for fish aggregation.

There are some limitations in the present study. Firstly, only one recorder was used, which made it impossible to determine the orientation of the sound source, i.e., we could not directly count the animals the recorder recorded. Then, shellfish aquaculture and fixed fishing net flourish in the Miaodao Archipelago. This was particularly the case in part III, part IV, and the area close to the islands, which hindered towing a recorder behind the survey boat. Thirdly, there is still the possibility that humpback, minke, and killer whales were missed due to our limited sampling efforts. For example, mammal-eating killer whales often travel or forage without discernibly echolocating [22]. In the future, PAM towing hydrophone array, long-term stationary PAM, and visual surveys, are recommended to be carried out to clarify the movement pattern of the humpback, minke, and killer whales, as well as the population status, size, age class composition, habitat range, and annual or seasonal present pattern of the East Asian finless porpoises in this water.

The conservation of cetaceans in the Miaodao Archipelago water is insufficient due to a previous lack of baseline knowledge. The findings of the present study underscore the importance of conducting further research, taking conservation measures to protect cetaceans in this area. Marine anthropogenic noise and fish resource management are key issues in protecting cetaceans [5]. Therefore, it is important to regulate human activities such as commercial shipping, oil extracting, and wind turbine construction in the Miaodao Archipelago water. The use of fishing methods such as bottom trawling and drift gillnets should be prohibited in this area in order to protect the marine ecosystem. There is a need for increased education initiatives in communities and schools to raise public awareness about marine mammal conservation. Establishing rescue organizations to save marine mammals injured in bycatch events is also important. In the future, it will be necessary to establish a marine protected area covering these regions.

## Figures and Tables

**Figure 5:**
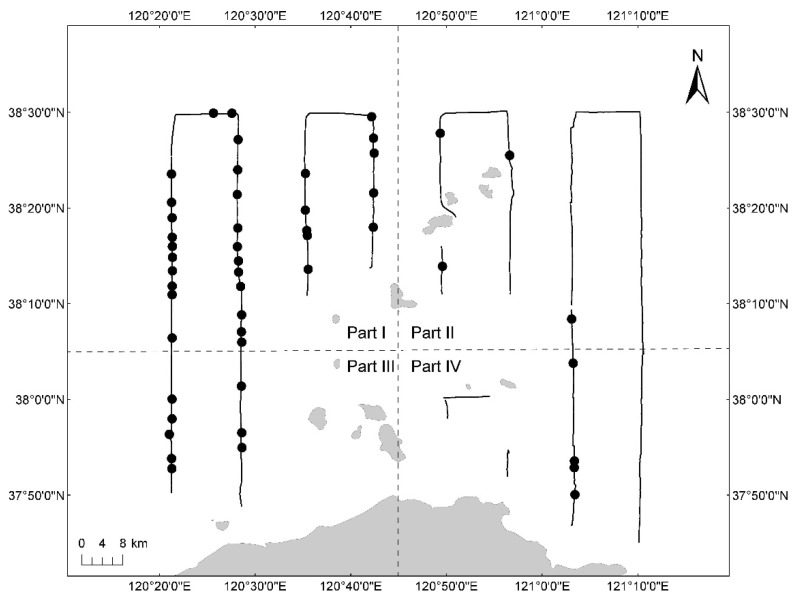
Survey routes (black line) and acoustic detection location (black dot) for the East Asian finless porpoises in Miaodao Archipelago waters in July 2022.

**Table 1:** Details of the results of the towed passive acoustic monitoring in the Miaodao Archipelago waters in May 2021, October 2021, and July 2022.

		Survey		
		May 2021	October 2021	July 2022
Part I	Detection	33	27	33
	Distance (km)	200.65	154.76	176.44
	Density (/km)	0.16	0.17	0.19
Part II	Detection	8	21	4
	Distance (km)	59.63	146.26	183.75
	Density (/km)	0.13	0.14	0.02
Part III	Detection	8	7	8
	Distance (km)	54.66	32.96	57.58
	Density (/km)	0.15	0.21	0.14
Part IV	Detection	7	9	4
	Distance (km)	41.55	89.54	86.99
	Density (/km)	0.17	0.10	0.05

**Table 2:** Details of the results of the stationary passive acoustic monitoring in Miaodao Archipelago waters in May 2021, October 2021, and July 2022.

Site	Monitoring Time	Detection Duration	Proportion
20210518	810 min	164 min	20.25%
20210519	820 min	805 min	98.18%
20210520	645 min	505 min	78.29%
20210522	2500 min	682 min	27.29%
20210524	662 min	221 min	33.33%
20210527	2040 min	30 min	1.45%
20211012	894 min	178 min	19.89%
20211013	768 min	339 min	44.16%
20211014	1001 min	0 min	0%
20211020	755 min	69 min	9.09%
20211021	1117 min	15 min	1.34%
20211022	736 min	15 min	2.01%
20220717	775 min	0 min	0%
20220718	645 min	86 min	13.28%
20220720	1352 min	40 min	2.94%
20220721	2180 min	0 min	0%
20220723	630 min	34 min	5.47%
20220724	615 min	79 min	12.90%

## Data Availability

The data presented in this study are available on request from the corresponding author.

## References

[1] Wang X.L., Zhang J. (2007). A nonlinear model for assessing multiple probabilistic risks: A case study in South five-island of Miaodao National Nature Reserve in China. J. Environ. Manag..

[2] Jefferson T.A., Webber M.A., Pitman R.L., Gorter U. (2015). Marine Mammals of the World.

[3] Cheng Z., Pine M.K., Li Y., Zuo T., Niu M., Wan X., Zhao X., Wang K., Wang J. (2021). Using local ecological knowledge to determine ecological status and threats of the East Asian finless porpoise, *Neophocaena asiaeorientalis sunameri*, in south Bohai Sea, China. Ocean. Coast Manag..

[4] Kimura S., Akamatsu T., Wang K., Wang D., Li S., Dong S., Arai N. (2009). Comparison of stationary acoustic monitoring and visual observation of finless porpoises. J. Acoust. Soc. Am..

[5] Cheng Z., Li Y., Pine M.K., Zuo T., Niu M., Wang J. (2023). Association between porpoise presence and fish choruses: Implications for feeding strategies and ecosystem-based conservation of the East Asian finless porpoise. Integr. Zool..

[6] Zimmer W.M.X. (2011). Passive Acoustic Monitoring of Cetacean.

[7] Barlow J., Cheeseman T., Trickey J.S. (2021). Acoustic detections of beaked whales, narrow-band high-frequency pulses and other odontocete cetaceans in the Southern Ocean using an autonomous towed hydrophone recorder. Deep. Sea Res. Part II Top. Stud. Oceanogr..

[8] Ross-Marsh E.C., Elwen S.H., Prinsloo A.S., James B.X., Gridley T. (2021). Singing in South Africa: Monitoring the occurrence of humpback whale (*Megaptera novaeangliae*) song near the Western Cape. Bioacoustics.

[9] Silber G.K. (1986). The relationship of social vocalizations to surface behavior and aggression in the Hawaiian humpback whale (*Megaptera novaeangliae*). Can. J. Zool..

[10] Tsujii K., Akamatsu T., Okamoto R., Mori K., Mitani Y., Umeda N. (2018). Change in singing behavior of humpback whales caused by shipping noise. PLoS ONE.

[11] Nikolich K., Towers J. (2018). Vocalizations of common minke whales (*Balaenoptera acutorostrata*) in an eastern North Pacific feeding ground. Bioacoustics.

[12] Oswald J.N., Au W.W., Duennebier F. (2011). Minke whale (*Balaenoptera acutorostrata*) boings detected at the Station ALOHA Cabled Observatory. J. Acoust. Soc. Am..

[13] Ford J.K. (1989). Acoustic behaviour of resident killer whales (*Orcinus orca*) off Vancouver Island, British Columbia. Can. J. Zool..

[14] Wellard R., Erbe C., Fouda L., Blewitt M. (2015). Vocalisations of Killer Whales (*Orcinus orca*) in the Bremer Canyon, Western Australia. PLoS ONE.

[15] Reyes Reyes M.V., Baumann-Pichering S., Simonis A., Melcón M.L., Trickey J., Hildebrand J., Iñíguez M. (2017). High-frequency modulated signals recorded off the Antarctic Peninsula area: Are killer whales emitting them?. Acoust. Aust..

[16] Li S., Wang D., Wang K., Akamatsu T., Ma Z., Han J. (2007). Echolocation click sounds from wild inshore finless porpoise (*Neophocaena phocaenoides sunameri*) with comparisons to the sonar of riverine *N. p. asiaeorientalis*. J. Acoust. Soc. Am..

[17] Wang P. (2012). Chinese Cetaceans.

[18] Chen D. (1991). Fishery Ecology of the Bohai Sea and the Yellow Sea.

[19] Dransfield A., Hines E., McGowan J., Holzman B., Nu N., Elliott M., Howar J., Jahncke J. (2014). Where the whales are: Using habitat modeling to support changes in shipping regulations within National Marine Sanctuaries in Central California. Endang. Species Res..

[20] Brough T., Rayment W., Slooten E., Dawson S. (2020). Spatiotemporal distribution of foraging in a marine predator: Behavioural drivers of hotspot formation. Mar. Ecol. Prog. Ser..

[21] Cheng Z., Yu G., Li Y., Zuo T., Niu M., Wang J. (2022). Distribution pattern of the East Asian finless porpoise in the Huanghe River estuary and its adjacent waters in spring. Oceanol. Limnol. Sin..

[22] Barrett-Lennard L.G., Ford J.K.B., Heise K.A. (1996). The mixed blessing of echolocation: Differences in sonar use by fish-eating and mammal-eating killer whales. Anim. Behav..

